# Supplementary material for: In vivo lensless microscopy via a phase mask generating diffraction patterns with high-contrast contours
Source: Nat Biomed Eng. 2022 Mar 7;6(5):617–28. doi: 10.1038/s41551-022-00851-z (PMC9142365; doi:10.1038/s41551-022-00851-z)
Supplement: Supplementary file 1 — Supplementary discussion, figures, tables, references and video captions. [file 41551_2022_851_MOESM1_ESM.pdf]

nature portfolio

https://doi.org/10.1038/s41551-022-00851-z

Supplementary information

In vivo lensless microscopy via a phase mask generating diffraction patterns with high-contrast contours

In the format provided by the authors and unedited

Contents

Supplementary discussion

Section 1. Modulation transfer function comparison among PSF designs

Section 2. Single capture three-dimensional imaging *in vivo*

Section 3. *In vivo* epifluorescence calcium imaging of whole animal with *Hydra vulgaris*

Supplementary figures, tables and videos

Supplementary Fig. 1 | Modulation transfer function (MTF) comparison among PSF designs used by lensless imaging systems.

Supplementary Fig. 2 | Experimentally captured contour PSF and calculated phase map for the mask.

Supplementary Fig. 3 | Bio-FlatScope with near-epi illumination with microprism and fiber-optic cable.

Supplementary Fig. 4 | Ca^{2+} imaging (GCaMP7b) and three-dimensional imaging (GFP) of *Hydra vulgaris in vivo*.

Supplementary Fig. 5 | Mouse brain slice imaging. Ground truth capture, raw capture by Bio-FlatScope, and Bio-FlatScope reconstruction of a mouse brain slice expressing GCaMP6f.

Supplementary Fig. 6 | Imaging through scattering medium.

Supplementary Fig. 7 | Simulation results of fluorescent beads with non-uniform irradiance.

Supplementary Fig. 8 | Full Bio-FlatScope field of view for *in vivo* mouse brain recordings.

Supplementary Fig. 9 | Comparison of Bio-FlatScope Ca^{2+} responses during stationary periods to stimulus-evoked responses in epifluorescence recordings.

Supplementary Fig. 10 | Spatiotemporal Ca^{2+} dynamics from epifluorescence recording.

Supplementary Fig. 11 | Simulation vs experimental result of USAF.

Supplementary Fig. 12 | Captured Bio-FlatScope point spread functions.

Supplementary Fig. 13 | Computational refocusing in mouse brain.

Supplementary Fig. 14 | Computational refocusing in human oral mucosa.

Supplementary Fig. 15 | Ca^{2+} responses from Bio-FlatScope recording across multiple ROIs.

Supplementary Fig. 16 | Oral mucosa imaging setup.

Supplementary Fig. 17 | Zoom-ins of Fig 3d,e.

Supplementary Fig. 18 | Extracting Ca^{2+} signals from epifluorescence recording with a 4× objective.

Supplementary Fig. 19 | Respective weights of the components of Bio-FlatScope.

Supplementary Table 1 l Comparison of Bio-FlatScope to miniaturized, head-mounted microscopes.

Supplementary Table 2 l Comparison of Bio-FlatScope to state-of-the-art lensless imaging technologies.

Supplementary Table 3 l Number of pixels and areas of each clustered ROI in Fig. 4.

Supplementary Video 1 l *Hydra vulgaris* GCamp7b.

Supplementary Video 2 l Mouse Cortex GCaMP6f.

References

Supplementary discussion

Section 1. Modulation transfer function comparison among PSF designs

In lensless imaging, the scene is encoded onto the sensor by convolution of the scene with a PSF. From convolution theorem, we can infer that for maximal information transfer, large and flat magnitude spectrum is desirable in the PSF. The deconvolution of PSF involves the inversion of the PSF's frequency spectrum, and low values of the magnitude spectrum can lead to amplification of noise.

We compared the Modulation Transfer Function (MTF) of our proposed contour PSF with PSFs designed for other lensless imaging systems (Supplementary Fig. 1). The MTF shown is computed as the radially averaged magnitude spectrum of the Fourier transform of the PSFs. The magnitude spectrum of our proposed contour PSF remains large for the entire frequency range, which indicates better invertibility characteristics.

Section 2. Single capture three-dimensional imaging *in vivo*

Using Bio-FlatScope we captured videos at 1 Hz of *Hydra* expressing GFP in interstitial cell lineage. We were then able to reconstruct these images in three dimensions (Supplementary Fig. 4a) during post-processing using a series of PSFs corresponding to different distances from the sensor in 50 µm increments. Here we show a 4 mm^3 volume reconstructed from a single capture. By contrast traditional microscopes typically require scanning spatially, axially, or both. This single-capture, three-dimensional imaging opens up the possibility for high-resolution, high-speed, volumetric imaging of whole organisms freely behaving over large FOVs.

Section 3. *In vivo* epifluorescence calcium imaging of whole animal with *Hydra vulgaris*

Given the high-speed volumetric imaging made possible by single-shot 3D imaging, we tested the ability of Bio-FlatScope to capture whole-animal dynamic calcium activity in *Hydra*. We began by imaging *Hydra vulgaris* expressing GCaMP7b in muscle cells [5]. We captured video of *Hydra* freely behaving at 2 Hz over a FOV >12 mm^2. In Supplementary Fig. 4b&c we show selected frames from a video of the reconstruction as well as the overall change in fluorescence over time ($\Delta F/F$). The recordings show strong calcium responses during contraction events as reported previously [6] (full video shown in Supplementary Video 1). Although movement of the animal can produce some noise in the $\Delta F/F$ signal, the deformable *Hydra* body makes it difficult to generate adaptive ROIs that move with the animal. Nevertheless, the bright synchronous calcium activity in the *Hydra* peduncle allows for fixed ROIs to effectively capture calcium dynamics [7,8]. Here the large FOV and 3D reconstructions help us capture movies of whole-animal calcium dynamics, which can be used by neuroscientists to study information processing in these small model organisms [9].

Supplementary figures and tables

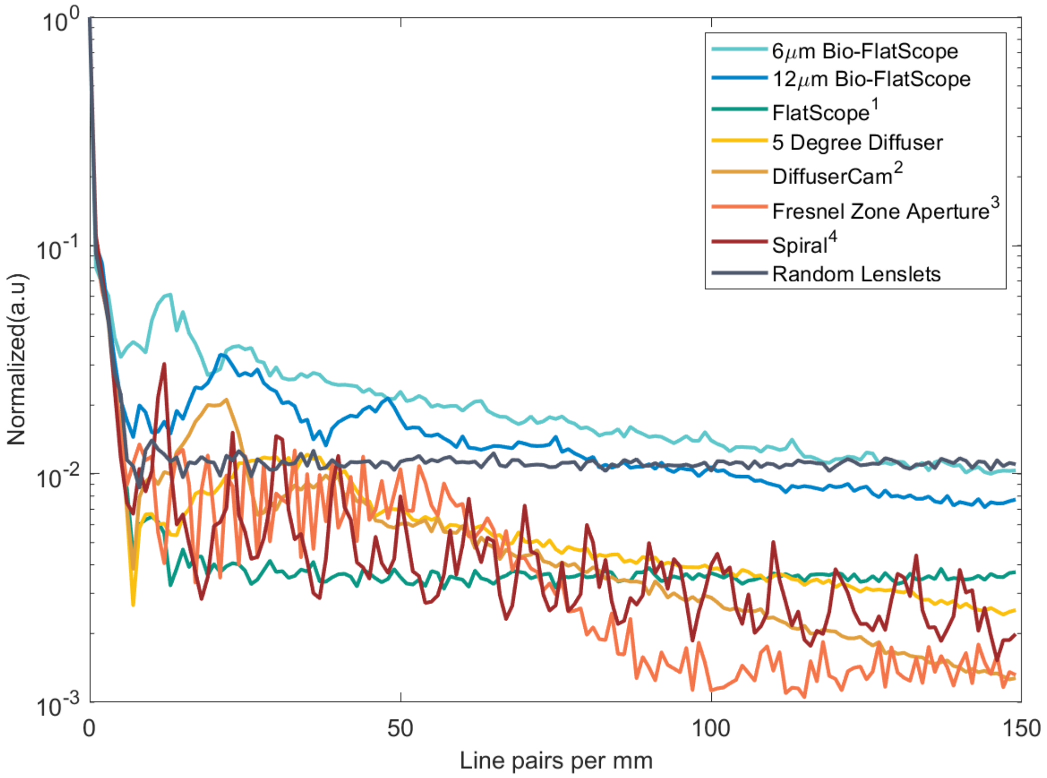

Supplementary Fig. 1 | Modulation transfer function (MTF) comparison among PSF designs used by lensless imaging systems. The PSFs compared are: Separable MSEQ used by FlatScope [1], 5 degree diffuser inspired, Diffuser used by DiffuserCam [2], Fresnel zone apertures (FZA) [3], Tessellated spiral [4] and Random lenslets

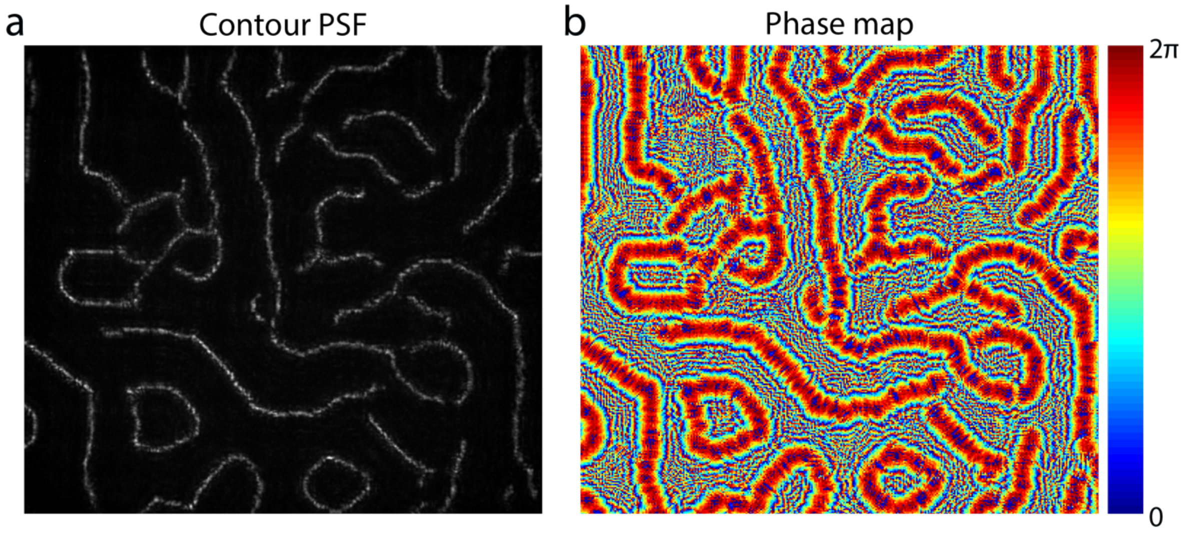

Supplementary Fig. 2 | Experimentally captured contour PSF and calculated phase map for the mask. a, Contour-based PSF design provides robustness to noise and capturing many directional filters. **b**, Phase map for the mask in order to produce the PSF on the image sensor.

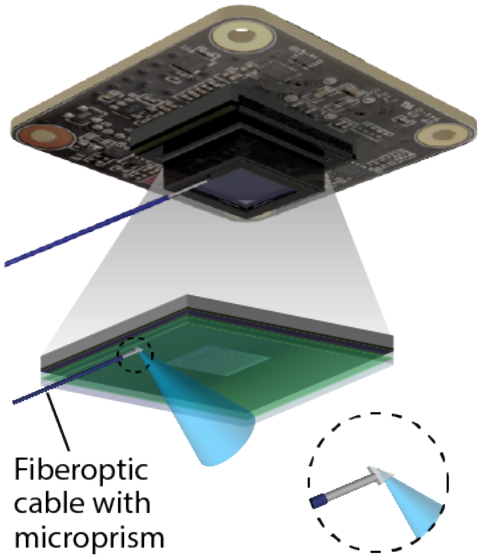

Supplementary Fig. 3 I Bio-FlatScope with near-epi illumination with microprism and fiber-optic cable.

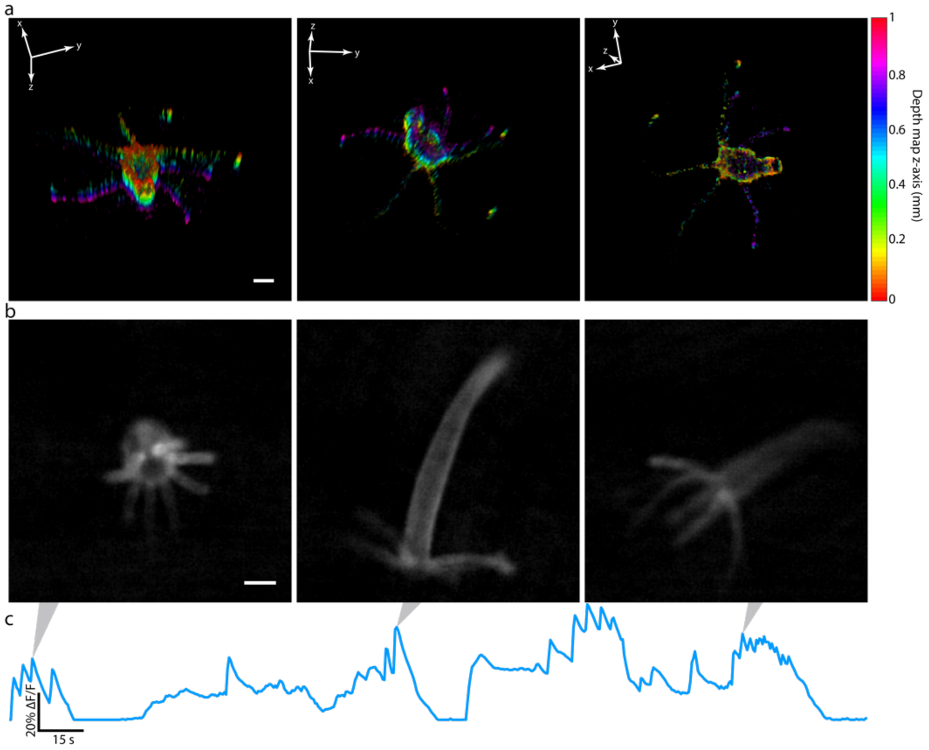

Supplementary Fig. 4 I Ca^{2+} imaging (GCaMP7b) and three-dimensional imaging (GFP) of *Hydra vulgaris in vivo*. a, Select angles of three-dimensional Bio-FlatScope reconstructions of *Hydra vulgaris* expressing GFP in the interstitial cell lineage. Colors represent relative depth location along the z-axis. Scale bar, 200 µm. **b**, Selected Bio-FlatScope reconstructed frames of video of *Hydra vulgaris* expressing GCaMP7b in muscle cells. Scale bar, 200 µm. **c**, ΔF/F traces showing the Ca^{2+} responses over a 5-minute recording. The ΔF/F in panel (**c**) is the change in the whole FOV at a single z-plane after reconstruction.

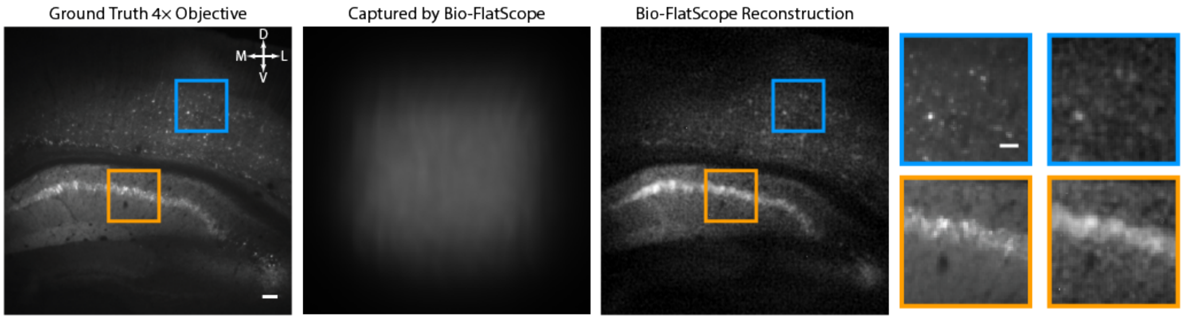

Supplementary Fig. 5 | Mouse brain slice imaging. Ground truth capture, raw capture by Bio-FlatScope, and Bio-FlatScope reconstruction of a mouse brain slice expressing GCaMP6f. The compass shows dorsal-D, ventral-D, medial-M and later-L directions. Scale bar, 100 µm. Far right shows zoom-in comparisons of ground truth and Bio-FlatScope reconstructions, respectively. Scale bar, 50 µm.

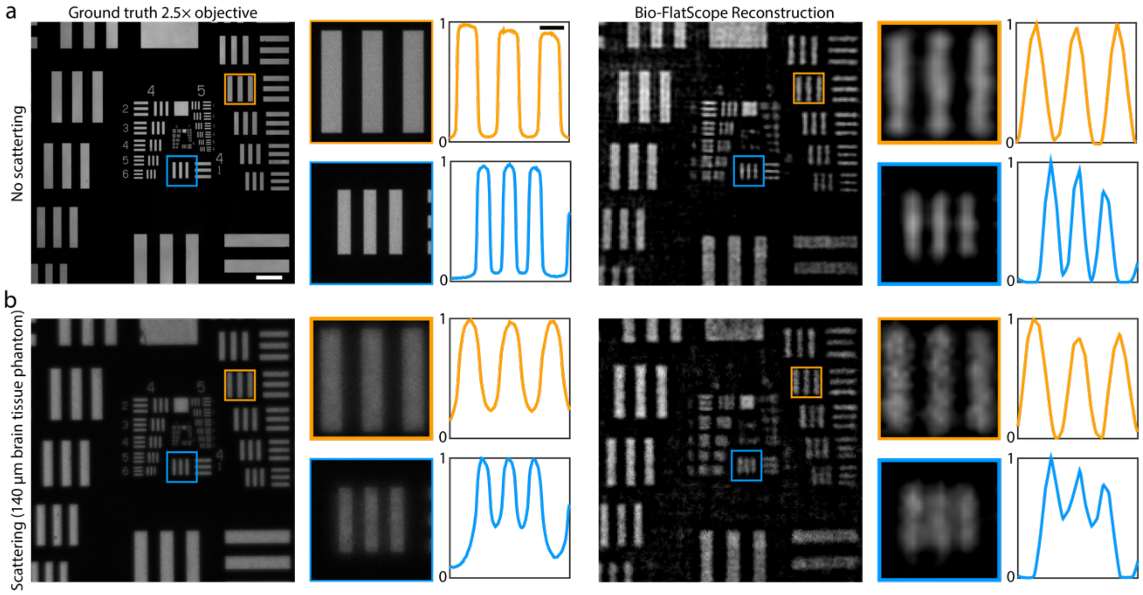

Supplementary Fig. 6 | Imaging through scattering medium. a, Ground truth (left, captured with a 2.5× objective and Bio-FlatScope reconstruction (right, captured at ~8.5 mm from the device) of USAF 1951 resolution target. Scale bar, 250 µm. Zoom-ins are shown for Group 3 element 3 and group 4 element 1. Scale bar, 50 µm. **b**, Ground truth (left, captured with a 2.5× objective) and Bio-FlatScope reconstruction (right, captured at ~8.5 mm from the device) of USAF 1951 resolution target captured through 140 µm of brain tissue phantom. Scale bar, 250 µm. Zoom-ins are shown for Group 3 element 3 and group 4 element 1. Scale bar, 50 µm.

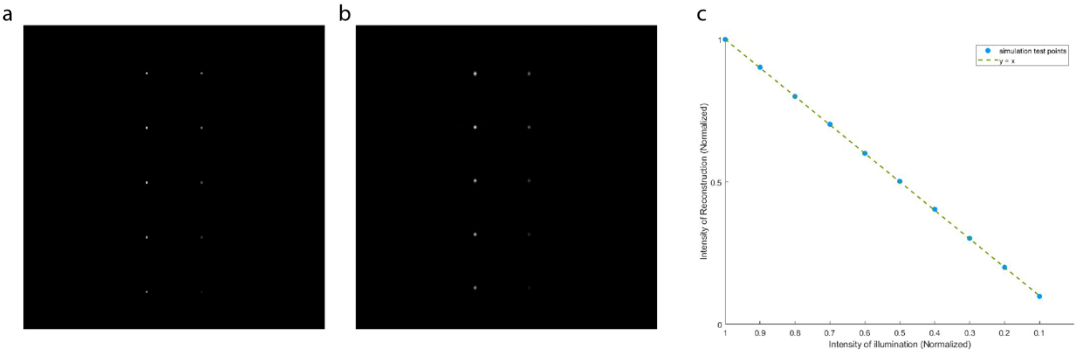

Supplementary Fig. 7 | Simulation results of fluorescent beads with non-uniform irradiance. a, Simulated target. Ten fluorescent beads in total (3×3 pixels, each pixel corresponding to 4 µm length as consistent with our experimental determined pixel size, i.e., each bead is 12 µm) with different illumination intensities (hence different irradiance) are simulated as the imaging target. **b**, Reconstruction result of the simulated target. **c**, Intensity plot of the reconstructed beads under different illumination intensities. We can see that the intensity of each reconstructed bead is linearly proportional to the intensity of illumination applied to it. This will allow us to perform renormalization of fluorescence intensity as is traditionally done for fluorescence microscopy [10].

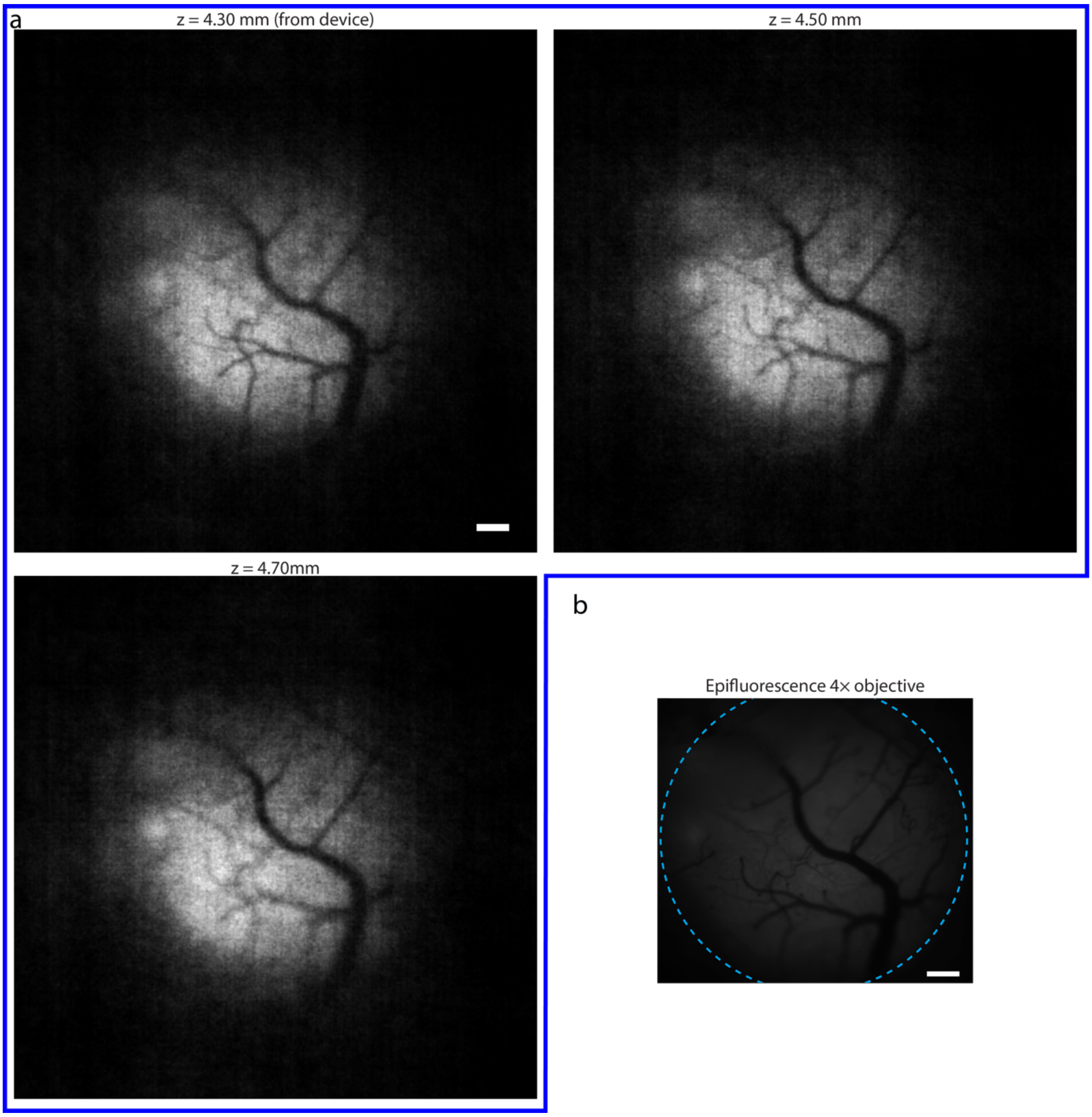

Supplementary Fig. 8 | Full Bio-FlatScope field of view for *in vivo* mouse brain recordings. a, Full FOV Bio-FlatScope reconstructions of mouse brain *in vivo* at 4.3 mm, 4.5 mm, and 4.7 mm from the device. Dark areas (around edges) are regions located outside of the cranial window. Scale bar, 250 µm. **b**, Epifluorescence image of same region of the mouse brain captured with a 4× microscope objective. Dashed cyan line indicates the outer edge of the cranial window. Scale bar, 250 µm.

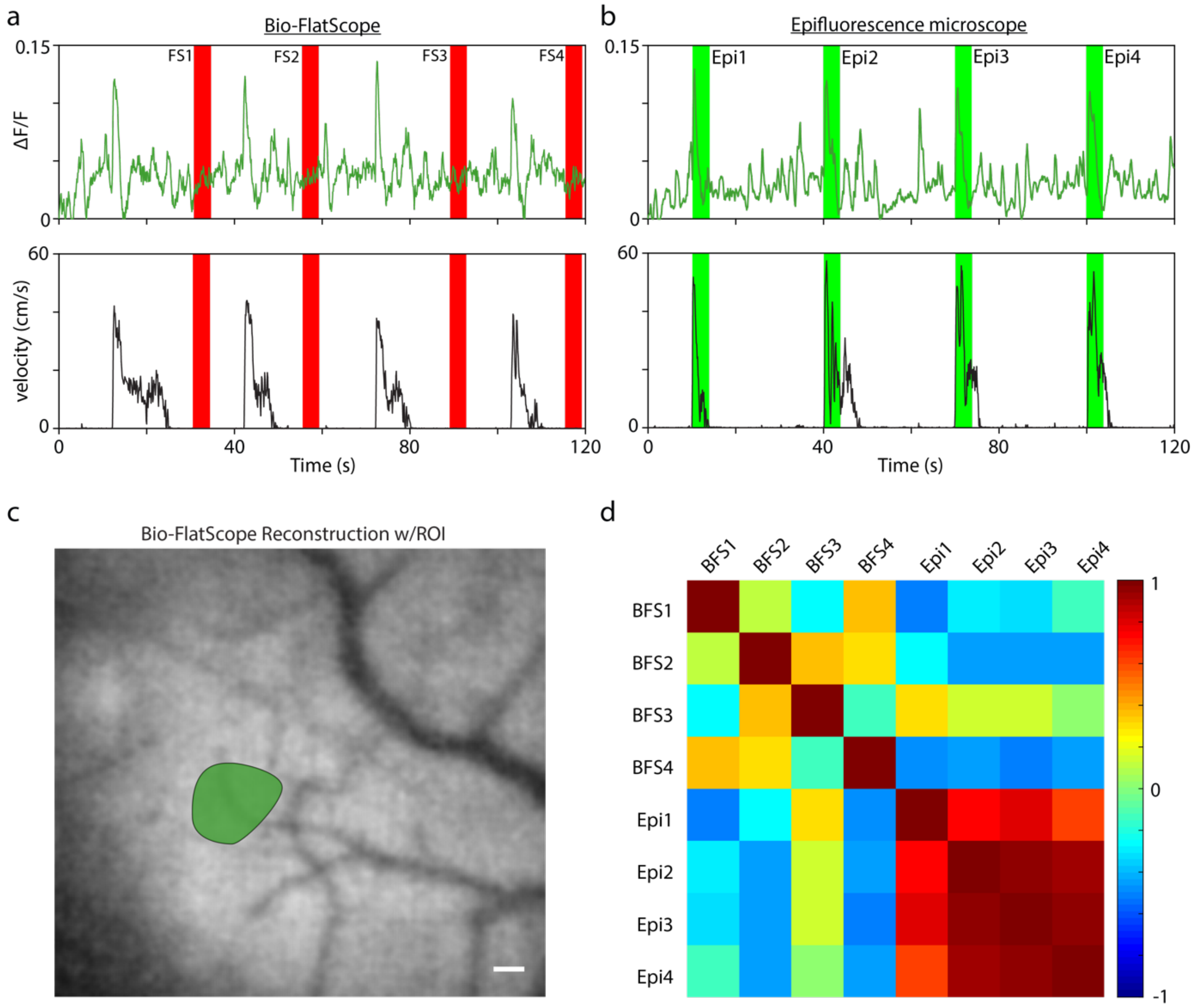

Supplementary Fig. 9 | Comparison of Bio-FlatScope Ca^{2+} responses during stationary periods to stimulus-evoked responses in epifluorescence recordings. a, ΔF/F trace and treadmill velocity for Bio-FlatScope during recording session. The 4-second windows are selected when the mouse is stationary (having a velocity <1 cm/s), shown in red. **b**, ΔF/F trace and treadmill velocity for epifluorescence during recording session. The rising edge of the 4-second windows in green correspond to the application of tactile stimuli. **c**, Bio-FlatScope reconstruction with a single ROI of high-activity marked. Scale bar, 100 µm. **d**, Correlation matrix comparing the 4-second windows of little to no movement (red) and stimulus response activity (green). (BFS - Bio-FlatScope, Epi-Epifluorescence microscope).

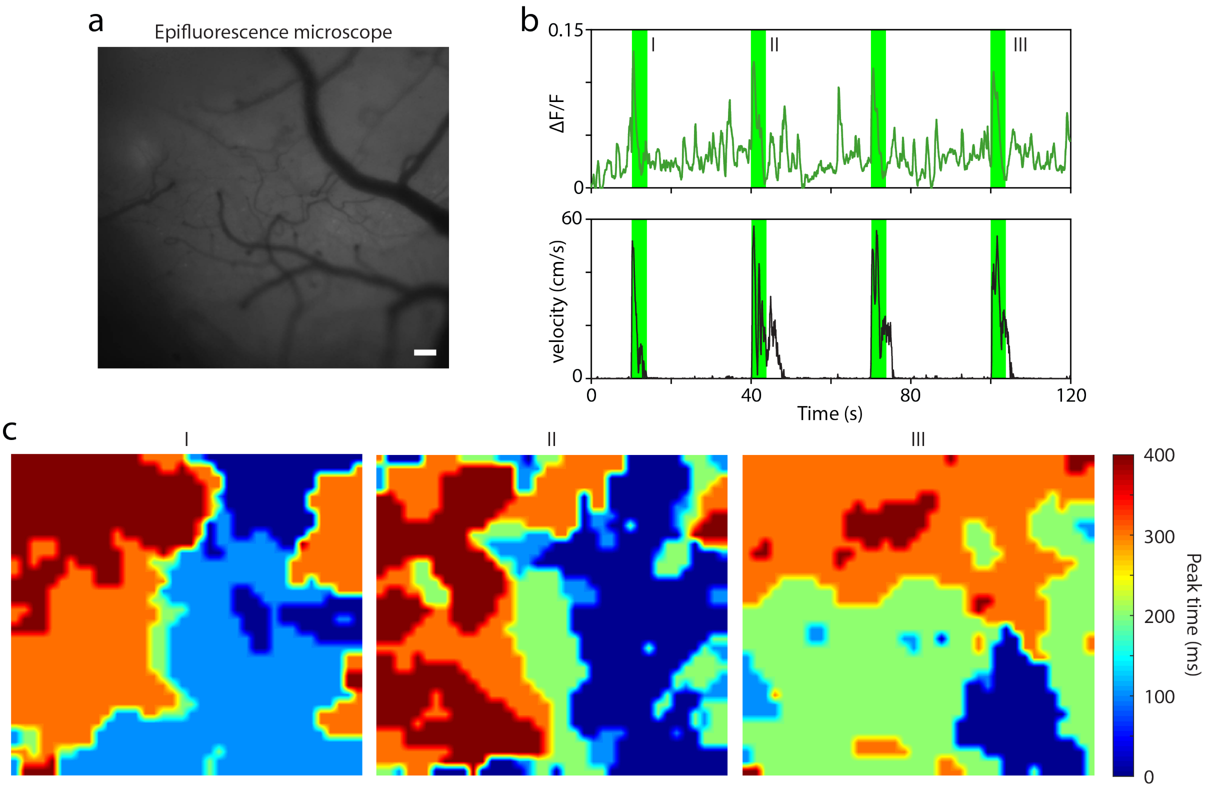

Supplementary Fig. 10 | Spatiotemporal Ca^{2+} dynamics from epifluorescence recording. a, Single frame from epifluorescence microscope capture of ROI. Scale bar, 100 µm. **b**, ΔF/F trace and treadmill velocity for epifluorescence during recording session. The rising edge of the 4-second windows in green correspond to the application of tactile stimuli. **c**, Heat maps for epifluorescence captures showing spatiotemporal Ca^{2+} dynamics time-aligned with stimuli (at **I**, **II**, and **III**). Colormap shows the time at which pixels have their peak response for ΔF/F during a 400 ms period.

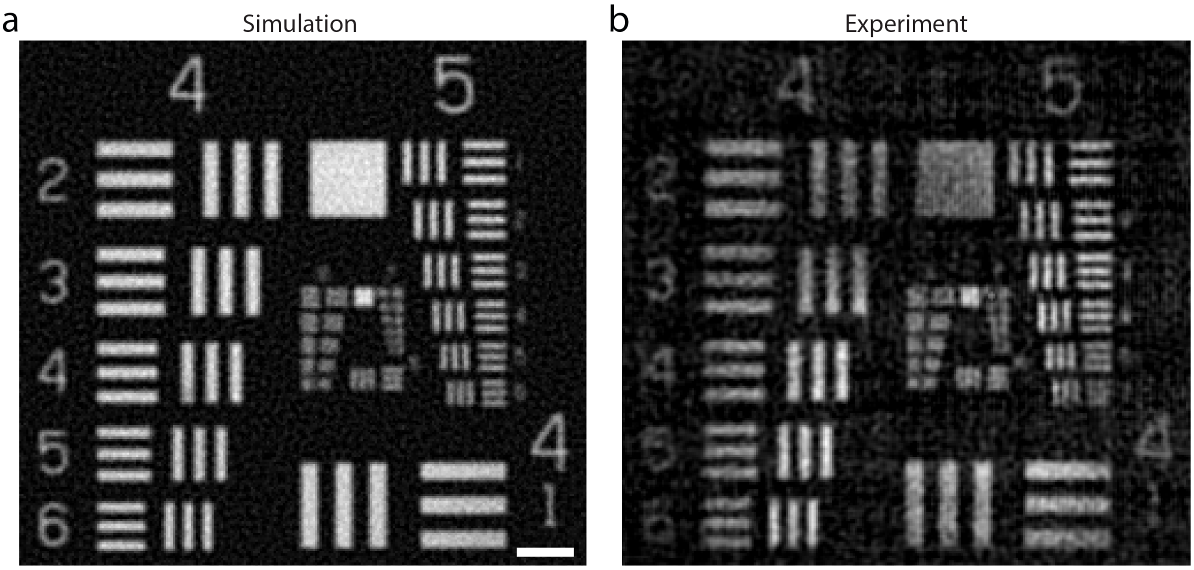

Supplementary Fig. 11 | Simulation vs experimental result of USAF. a, Simulated Bio-FlatScope reconstruction of USAF target at 4.15 mm from device. Scale bar, 100 µm. **b**, Experimental Bio-FlatScope reconstruction of USAF target at the same distance from device. The simulation result shows a very close match to the experimental result with group 5 element 6 being resolved.

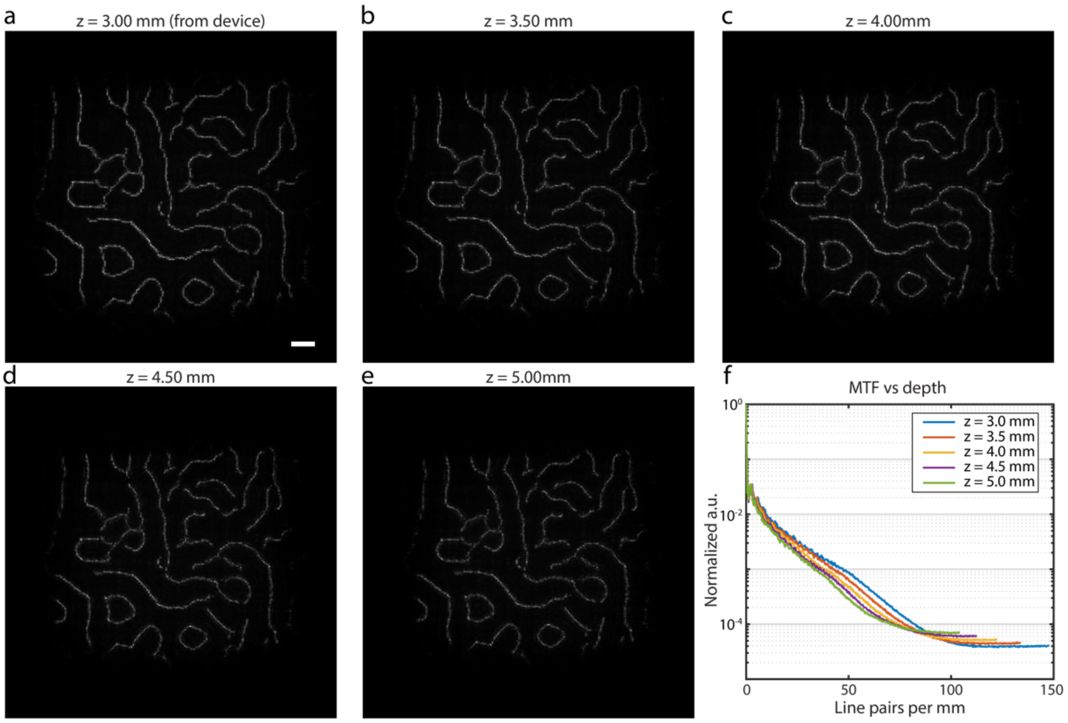

Supplementary Fig. 12 | Captured Bio-FlatScope point spread functions. Bio-FlatScope PSFs captured by imaging a 10 µm fluorescent microsphere (see *Methods*) at different depth planes of **a**, 3.00 mm. **b**, 3.50 mm. **c**, 4.00 mm. **d**, 4.50 mm. **e**, 5.00 mm. Scale bar, 100 µm. **f**, Computed MTF from PSFs at different depths from Bio-FlatScope. We observe that the resolution decreases with increasing depth, as seen from the shifting of MTF curves to the left.

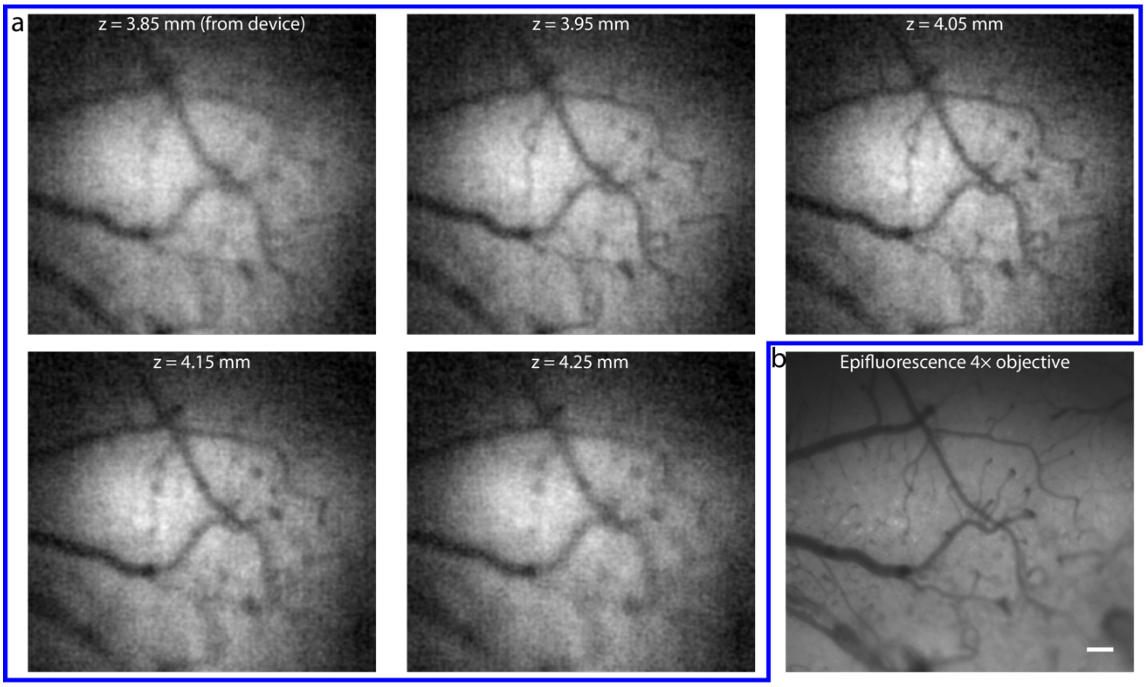

Supplementary Fig. 13 | Computational refocusing in mouse brain. a, Bio-FlatScope reconstructions for five different depth planes. At 4.05 mm, the image becomes sharpest for the FOV. **b**, Epifluorescence image for the same FOV. Scale bar, 100 µm.

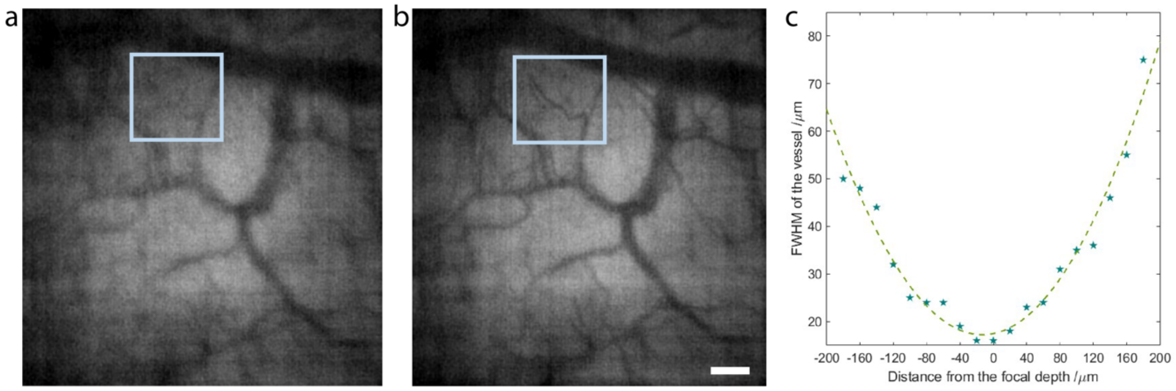

Supplementary Fig. 14 | Computational refocusing in human oral mucosa. a, Bio-FlatScope reconstructions at 2.88 mm depth. The image is out of focus and some of the small features cannot be reconstructed. **b**, Bio-FlatScope reconstructions at 3.04 mm depth. The image becomes sharp and small vessels can be reconstructed at high contrast. Scale bar, 100 µm **c**, FWHM of the intensity across the small vessel in the blue square shown in panel (**a**) and (**b**) at different imaging depths.

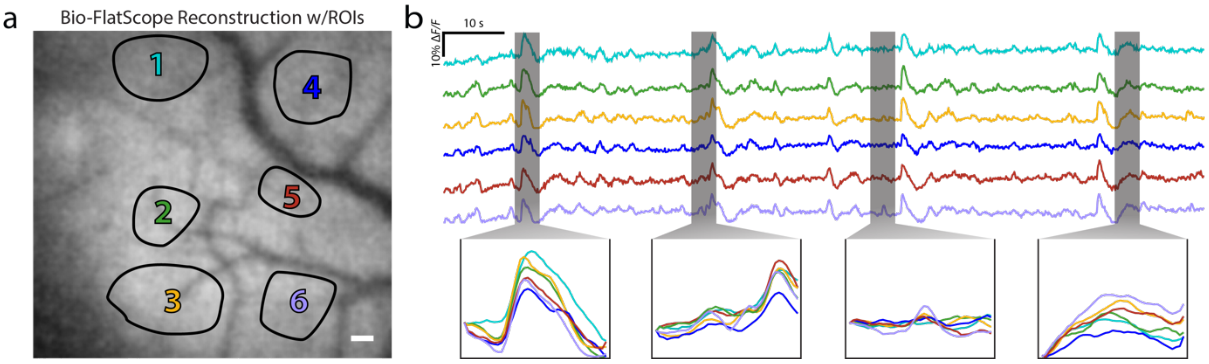

Supplementary Fig. 15 | Ca^{2+} responses from Bio-FlatScope recording across multiple ROIs. a, Bio-FlatScope reconstruction showing multiple ROIs. Scale bar 100 µm. **b**, ΔF/F traces for the multiple regions with zoom-ins showing differences in activity across ROIs.

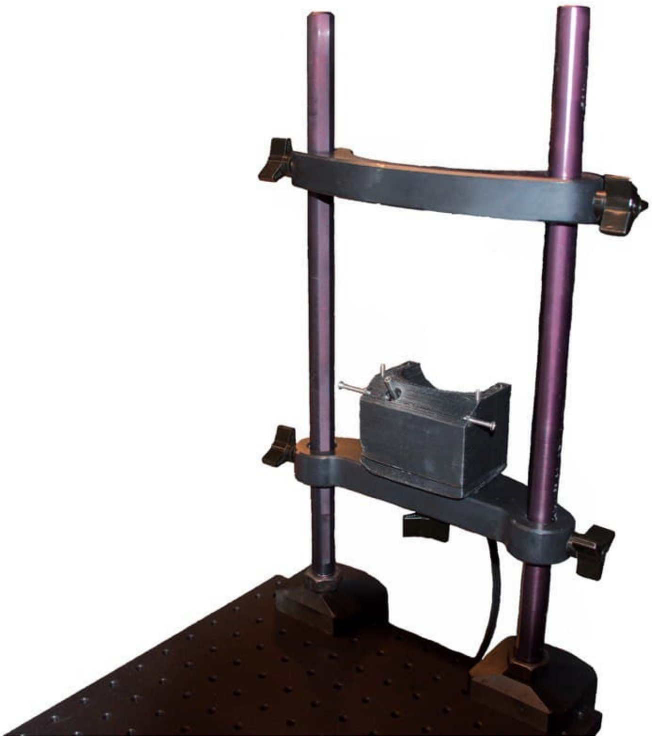

Supplementary Fig. 16 | Oral mucosa imaging setup. The setup includs a chin-head rest and a liquid light guide connected to a tabletop LED source for illumination. This photo shows how the liquid light guide is mounted in the chin rest.

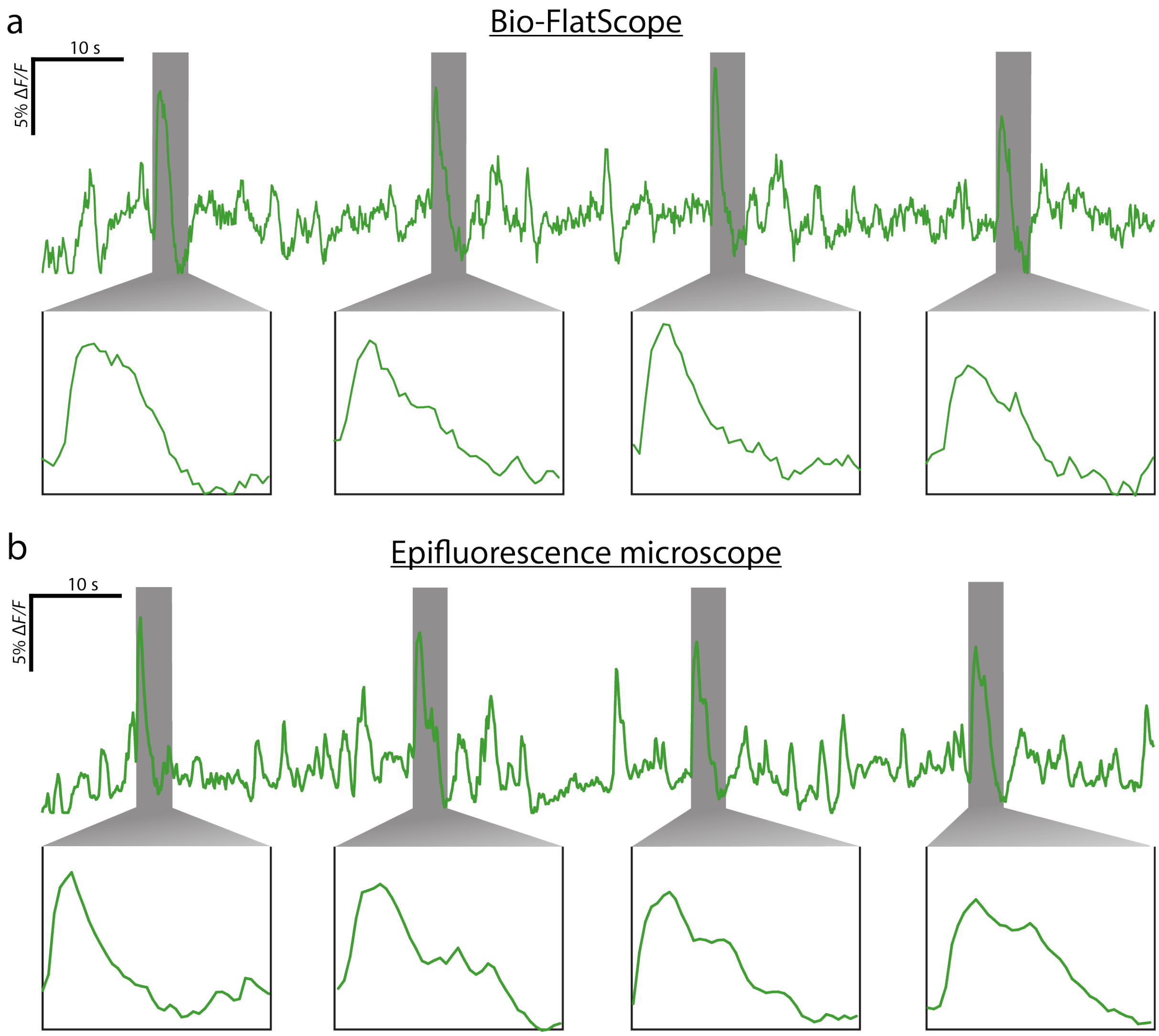

Supplementary Fig. 17 I Zoom-ins of Fig 3d,e. These zoom-ins show ΔF/F corresponding to the stimulation events for comparison of Bio-FlatScope and epifluorescence. Note that these measurements were not taken simultaneously, so we do not expect an exactly identical response.

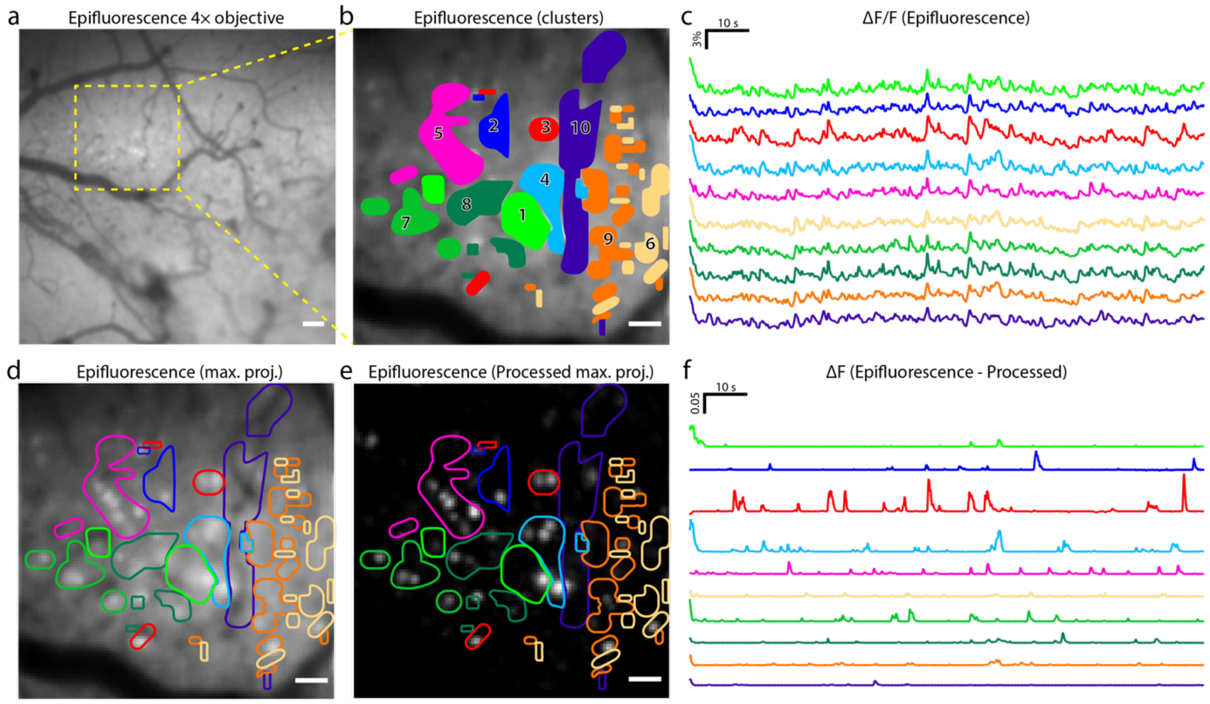

Supplementary Fig. 18 l Extracting Ca^{2+} signals from epifluorescence recording with a 4× objective. a, Cropped region of a single frame with a high-activity region shown by a dashed box. Scale bar, 100 µm. **b**, Zoom-in on the region of high-activity with overlay of clusters determined (from Bio-FlatScope data) through post-processing using RPCA and k-means. Scale bar, 50 µm. **c**, ΔF/F traces from the video over during two-minute recording corresponding to the clusters. **d**, Maximum projection from epifluorescence recording of same high activity region with overlay of clusters. Scale bar 50 µm. **e**, Maximum intensity projection of epifluorescence recording data processed using RPCA with overlay of clusters. Scale bar 50 µm. **f**, ΔF traces from epifluorescence recording after post-processing using RPCA. Note that the clusters shown here were found using the Bio-FlatScope data.

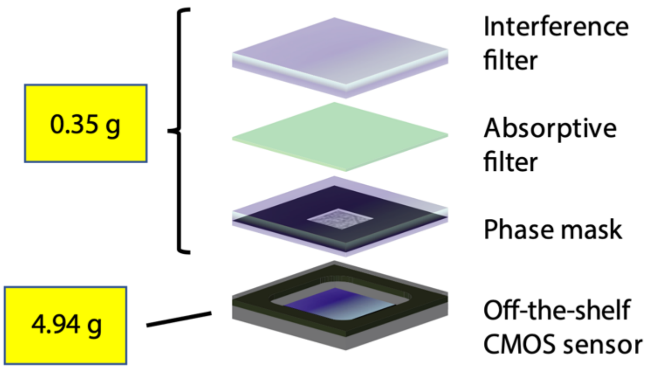

Supplementary Fig. 19 l Respective weights of the components of Bio-FlatScope. Note that the off-the-shelf CMOS sensor contributes to the majority of the total weight, suggesting that Bio-FlatScope can be applied to free-living rodents by miniaturizing the PCB supporting the image sensor as has been reported for other miniature head-mounted microscopes.

Supplementary Table 1 I Comparison of Bio-FlatScope to miniaturized, head-mounted microscopes.

Microscope	Lateral resolution	Field of view
Bio-FlatScope [a]	~8.8 µm	~16.2 mm^2
Integrated miniature microscope [11]	~2.5 µm	0.48 mm^2
Fiber bundle microscope [12]	~4.9 µm	~0.3 mm^2
Two-photon miniaturized microscope [13]	0.64 µm	~0.03 mm^2
Miniaturized light-field microscope [14]	6 µm	~0.42 mm^2
Miniscope3D [16]	2.76 µm	0.9 × 0.7 × 0.39 mm^3
Mini-mScope [18]	39.36 ~ 55.68 µm	80 mm^2

[a] Note that for Bio-FlatScope to reach the same < 3g weight as head-mounted microscopes, the sensor PCB will need to be miniaturized like the miniature lens-based microscopes shown here.

Supplementary Table 2 I Comparison of Bio-FlatScope to state-of-the-art lensless imaging technologies.

Microscope	Any *in vivo* imaging	*In vivo* imaging of mammalian tissue	Working distance [a]	Lateral resolution	Axial resolution	Field of view
Bio-FlatScope	Yes	Yes	~4 mm	~8.8 µm	50 µm	~4 × 4 mm^2
FlatScope [1]	No	No	200 µm	7 µm	15 µm	2.5 × 2.5 mm^2
On-chip microscope [15]	Yes	No	1.5-3 mm	8 µm	50 µm	2 × 1.5 mm^2 [b]
DiffuserCam [2] [c1]	No	No	20 mm	45 µm	336 µm	36 × 20 mm^2 [c2]
CM^2 [17]	No	No	12 mm	7 µm	200 µm	8 × 7 mm^2

[a] The working distance is defined as the distance at which the parameters in the table are characterized.

[b] The authors did not explicitly mention their FOV in the manuscript. From their fluorescence bead imaging results shown in Fig 8 we estimated the FOV to be 2 × 1.5 mm^2.

[c1] This work reports a camera with 45 micron spatial resolution and long working distance rather than a microscope as described here. Nevertheless, Figure 1 of our manuscript compares our Bio-FlatScope to a diffuser-based device.

[c2] The FOV of this work is reported as angle instead of length, which is typical for cameras. The authors report their FOV as half-angle 42° in x and 30.5° in y, corresponding to 36 mm in x and 23.6 mm in y at 20 mm working distance.

Supplementary Table 3 | Number of pixels and areas of each clustered ROI in Fig. 4.

Cluster number	Number of pixels	Area (mm^2)
1	316	0.0052
2	144	0.0024
3	88	0.0014
4	300	0.0049
5	316	0.0052
6	308	0.0050
7	208	0.0034
8	288	0.0047
9	444	0.0072
10	700	0.0114

References

1. Adams, J. K. *et al.* Single-frame 3D fluorescence microscopy with ultraminiature lensless FlatScope. *Sci. Adv.* **3**, e1701548 (2017).

2. Antipa, N. *et al.* DiffuserCam: lensless single-exposure 3D imaging. *Optica* **5**, 1, 1-9 (2018).

3. Tajima, K., Shimano, T., Nakamura, Y., Sao, M. & Hoshizawa, T. Lensless light-field imaging with multi-phased fresnel zone aperture. 2017 IEEE International Conference on Computational Photography. doi:10.1109/ICCPHOT.2017.7951485

4. Stork, D. G. & Gill, P. R. Optical, Mathematical, and Computational Foundations of Lensless Ultra-Miniature Diffractive Imagers and Sensors. *International Journal on Advances in Systems and Measurements* **7**, 201–208 (2014).

5. Badhiwala, K. N., Primack, A. S., Juliano, C. E. & Robinson, J. T. Multiple nerve rings coordinate *Hydra* mechanosensory behavior. *eLife* **10** (2020).

6. Szymanski, J. R. & Yuste, R. Mapping the whole-body muscle activity of *Hydra vulgaris*. *Curr. Biol.* **29**, 11, 1807-1817 (2019).

7. Badhiwala, K. N., Gonzales, D. L., Vercosa, D. G., Avants, B. W. & Robinson, J. T. Microfluidics for electrophysiology, imaging, and behavioral analysis of *Hydra*. *Lab. Chip* **18**, 2523–2539 (2018).

8. Tzouanas, C. N., Kim, S., Badhiwala, K. N., Avants, B. W. & Robinson, J. T. Thermal stimulation temperature is encoded as a firing rate in a *Hydra* nerve ring. *bioRxiv* 787648 (2019).

9. Dombeck, D. A., Graziano, M. S. & Tank, D. W. Functional Clustering of Neurons in Motor Cortex Determined by Cellular Resolution Imaging in Awake Behaving Mice. *J. Neurosci.* **29**, 13751–13760 (2009).

10. Nguyen, J. P. *et al.* Whole-brain calcium imaging with cellular resolution in freely behaving Caenorhabditis elegans. *Proc. Natl. Acad. Sci. U. S. A.* **113**, E1074–E1081 (2016).

11. Ghosh, K. K. *et al.* Miniaturized integration of a fluorescence microscope. *Nat. Methods* **8**, 871–878 (2011).

12. Kim, M., Hong, J., Kim, J. & Shin, H. Fiber bundle-based integrated platform for wide-field fluorescence imaging and patterned optical stimulation for modulation of vasoconstriction in the deep brain of a living animal. *Biomed. Opt. Express* **8**, 2781 (2017).

13. Zong, W. *et al.* Fast high-resolution miniature two-photon microscopy for brain imaging in freely behaving mice. *Nat Methods* **14**, 713–719 (2017).

14. Skocek, O. *et al.* High-speed volumetric imaging of neuronal activity in freely moving rodents. *Nat. Methods* **15**, 429–432 (2018).

15. Kuo, G. *et al.* On-chip fluorescence microscopy with a random microlens diffuser. *Opt. Express* **28**, 8384-8399 (2020)

16. Yanny, K. *et al.* Miniscope3D: optimized single-shot miniature 3D fluorescence microscopy. *Light Sci. Appl.* **9**, (2020).

17. Xue, Y., Davison, I. G., Boas, D. A. & Tian, L. 3D Fluorescence Imaging with a Computational Mesoscope. *Sci. Adv.* 1–16 (2020). doi:10.1109/IPC47351.2020.9252229

18. Rynes, M. L. *et al.* Miniaturized head-mounted microscope for whole-cortex mesoscale imaging in freely behaving mice. *Nat. Methods* **18**, 417–425 (2021).
