# Supplementary material for: In vivo lensless microscopy via a phase mask generating diffraction patterns with high-contrast contours
Source: Nat Biomed Eng. 2022 Mar 7;6(5):617–28. doi: 10.1038/s41551-022-00851-z (PMC9142365; doi:10.1038/s41551-022-00851-z)
Supplement: Supplementary file 2 — Reporting Summary [file 41551_2022_851_MOESM2_ESM.pdf]

Corresponding author(s): Jacob T. Robinson and Ashok Veeraraghavan

Last updated by author(s): Jan 24, 2022

Reporting Summary

Nature Portfolio wishes to improve the reproducibility of the work that we publish. This form provides structure for consistency and transparency in reporting. For further information on Nature Portfolio policies, see our Editorial Policies and the Editorial Policy Checklist.

Statistics

For all statistical analyses, confirm that the following items are present in the figure legend, table legend, main text, or Methods section.

n/a	Confirmed	
☐	☒	The exact sample size (n) for each experimental group/condition, given as a discrete number and unit of measurement
☐	☒	A statement on whether measurements were taken from distinct samples or whether the same sample was measured repeatedly
☒	☐	The statistical test(s) used AND whether they are one- or two-sided<br>*Only common tests should be described solely by name; describe more complex techniques in the Methods section.*
☒	☐	A description of all covariates tested
☒	☐	A description of any assumptions or corrections, such as tests of normality and adjustment for multiple comparisons
☐	☒	A full description of the statistical parameters including central tendency (e.g. means) or other basic estimates (e.g. regression coefficient) AND variation (e.g. standard deviation) or associated estimates of uncertainty (e.g. confidence intervals)
☒	☐	For null hypothesis testing, the test statistic (e.g. F, t, r) with confidence intervals, effect sizes, degrees of freedom and P value noted<br>*Give P values as exact values whenever suitable.*
☒	☐	For Bayesian analysis, information on the choice of priors and Markov chain Monte Carlo settings
☒	☐	For hierarchical and complex designs, identification of the appropriate level for tests and full reporting of outcomes
☒	☐	Estimates of effect sizes (e.g. Cohen's d, Pearson's r), indicating how they were calculated

Our web collection on statistics for biologists contains articles on many of the points above.

Software and code

Policy information about availability of computer code

Data collection	MATLAB. Imaging Source IC Capture. Thorlabs Thorcam.
Data analysis	The custom MATLAB codes for Bio-FlatScope image reconstruction are available on GitHub at https://github.com/JiminWu/Bio-FlatScope. Other custom MATLAB codes, including Bio-FlatScope capture, simulation and RPCA analysis, are available from the corresponding authors on request.

For manuscripts utilizing custom algorithms or software that are central to the research but not yet described in published literature, software must be made available to editors and reviewers. We strongly encourage code deposition in a community repository (e.g. GitHub). See the Nature Portfolio guidelines for submitting code & software for further information.

Data

Policy information about availability of data

All manuscripts must include a data availability statement. This statement should provide the following information, where applicable:

- Accession codes, unique identifiers, or web links for publicly available datasets
- A description of any restrictions on data availability
- For clinical datasets or third party data, please ensure that the statement adheres to our policy

The main data supporting the results in this study are available within the paper and its Supplementary Information. Sample data are available on GitHub at https://github.com/JiminWu/Bio-FlatScope. The raw and analysed datasets generated during the study are too large to be publicly shared, yet they are available for research purposes from the corresponding authors on reasonable request. The raw healthy volunteer data are available from the corresponding authors, subject to approval from Rice University's Institutional Review Board.

Field-specific reporting

Please select the one below that is the best fit for your research. If you are not sure, read the appropriate sections before making your selection.

☒ Life sciences ☐ Behavioural & social sciences ☐ Ecological, evolutionary & environmental sciences

For a reference copy of the document with all sections, see nature.com/documents/nr-reporting-summary-flat.pdf

Life sciences study design

All studies must disclose on these points even when the disclosure is negative.

Sample size	Human oral-mucosa imaging: Past studies have shown that microvessel-density variation is not significant among healthy human subjects. The use of 5 participants and 10 regions per participant were determined to sufficiently account for the variation of vessel counts within the field of view of each capture, and to show the vessel-counting correlation between 4x objective and Bio-FlatScope.
Data exclusions	No data were excluded from the analyses.
Replication	We imaged multiple regions of oral mucosa for each participant. Results are consistent for all images taken.
Randomization	This is not relevant to the study, as the conclusion of the microvessel-density correlation was drawn by the comparison of data collected from the same participant by two different methods.
Blinding	Blinding is not relevant to the study, as the conclusions drawn from the experiments are not dependent on the subject's awareness of being selected.

Reporting for specific materials, systems and methods

We require information from authors about some types of materials, experimental systems and methods used in many studies. Here, indicate whether each material, system or method listed is relevant to your study. If you are not sure if a list item applies to your research, read the appropriate section before selecting a response.

Materials & experimental systems

n/a	Involved in the study	
☒	☐	Antibodies
☒	☐	Eukaryotic cell lines
☒	☐	Palaeontology and archaeology
☐	☒	Animals and other organisms
☐	☒	Human research participants
☒	☐	Clinical data
☒	☐	Dual use research of concern

Methods

n/a	Involved in the study	
☒	☐	ChIP-seq
☒	☐	Flow cytometry
☒	☐	MRI-based neuroimaging

Animals and other organisms

Policy information about studies involving animals; ARRIVE guidelines recommended for reporting animal research

Laboratory animals	Hydra vulgaris, transgenetic, expressing either GFP in interstitial cell lineage (GFP, Neurons) or GCaMP7b in ectodermal muscle cells (GCaMP7b, Ecto).<br><br>Wild-type C57BL/6 mice (n = 7) from Charles River Laboratories were used for this study. All animals (n = 7) were injected with the adeno-associated viral vector AAV9.CamKII.GCaMP6f.WPRE.SV40 (Penn Vector Core).
Wild animals	The study did not involve wild animals.
Field-collected samples	The study did not involve samples collected from the field.
Ethics oversight	All experimental procedures were approved by the Institutional Animal Care and Use Committee at Rice University, and followed the guidelines of the National Institute of Health.

Note that full information on the approval of the study protocol must also be provided in the manuscript.

Human research participants

Policy information about studies involving human research participants

Population characteristics	All human research participants recruited in the study were 18 years or older, with no underlying health conditions (more specifically, not infected by diseases that affect microvessel density in the oral mucosa).
Recruitment	Participants were recruited within Rice University. No potential bias may be present that are likely to impact the results of the study, as past studies have shown that microvessel-density variation is not significant among healthy human subjects.
Ethics oversight	All volunteers provided health pre-screen information to reduce the risk of COVID spread. The study was in accord with the protocol approved by the Institutional Review Board at Rice University. Written informed consent was obtained from all volunteers prior to imaging.

Note that full information on the approval of the study protocol must also be provided in the manuscript.
